# MiR-146b is down-regulated during the chondrogenic differentiation of human bone marrow derived skeletal stem cells and up-regulated in osteoarthritis

**DOI:** 10.1038/srep46704

**Published:** 2017-04-24

**Authors:** Emma Budd, María C. de Andrés, Tilman Sanchez-Elsner, Richard O. C. Oreffo

**Affiliations:** 1 Bone and Joint Research Group, Centre for Human Developmental, Stem Cells and Regeneration, Faculty of Medicine, University of Southampton, Southampton, SO16 6YD, UK; 2 Junk RNA group, Clinical and Experimental Sciences, Faculty of Medicine, University of Southampton, Southampton, SO16 6YD, UK.

## Abstract

Articular cartilage injury can result in chondrocyte loss and diminishment of specialised extracellular matrix, which can progress to an osteoarthritic (OA) phenotype. Stem cells have emerged as a favourable approach for articular cartilage regeneration. Identification of miRNAs which influence stem cell fate offers new approaches for application of miRNAs to regenerate articular cartilage. Skeletal stem cells (SSCs) isolated from human bone marrow were cultured as high density micromass’ using TGF-β3 to induce chondrogenesis. qPCR and TaqMan qPCR were used to assess chondrogenic gene and miRNA expression. Target prediction algorithms identified potential targets of miR-146b. Transient transfection with miR-146b mimic and western blotting was used to analyse SOX5. Human OA articular chondrocytes were examined for miR-146b expression. Chondrogenic differentiation of human bone marrow derived SSCs resulted in significant down-regulation of miR-146b. Gain of miR-146b function resulted in down-regulation of SOX5. MiR-146b expression was up-regulated in OA chondrocytes. These findings demonstrate the functional role of miR-146b in the chondrogenic differentiation of human bone marrow derived SSCs. MiR-146b may play a role in the pathophysiology of OA. Application of miR-146b combined with stem cell therapy could enhance regeneration of cartilaginous tissue and serve as a potential therapeutic target in the treatment of OA.

Osteoarthritis (OA) is a prevalent chronic disease in an increasing ageing population, with 49% of women and 42% of men aged over 75 years requiring treatment for OA^1^. Radiological evidence indicates OA of the knee is the most prevalent cause of immobility^2^ and OA is associated with significant socio-economic costs. In the UK, annual medical costs associated with OA have been calculated at £320 million^3^. OA can be described as a heterogeneous group of conditions which result in joint signs and symptoms associated with changes to bone at joint margins and defective integrity of articular cartilage; degeneration of the articular cartilage and subchondral bone^4^. The exact causes of OA remain unknown and a number of confounding factors may initiate disease progression including injury, obesity and joint loading due to physical activity^5^. Articular cartilage is avascular, aneural and alymphatic, with embedded non-proliferating and non-migratory chondrocytes present within a specialised extracellular matrix (ECM), all factors likely contribute to the limited capacity of articular cartilage for intrinsic healing and repair following trauma. Cartilage injury as a result of torsion or intensive axial load and shear stress is likely to result in degenerative changes leading to the onset of OA^6^,^7^. In a study of athletes with isolated chondral lesions, initially most of the cohort did not require treatment. After 14 years a number of the athletes displayed a reduction of the joint space, indicating that while the initial chondral lesions were asymptomatic, degradation of the articular cartilage ensued leading to permanent knee damage^8^. Cartilage damage is likely to be proceeded by long term articular cartilage deterioration and OA^7^.

Repair of an initial articular cartilage defect could limit the subsequent articular cartilage deterioration and onset of OA. The immuno-modulatory and differentiation properties of SSCs make them a viable and promising cell source to repair cartilage^9^, the ability to direct SSCs down the chondrogenic lineage is a propitious option for articular cartilage regeneration. The therapeutic effect of SSCs administration to articular cartilage defects in patients has been previously reported. Nejadnik *et al*. found that patients administered with bone marrow stem cells into chondral lesions displayed better physical chondrocyte implantation^10^. Transplantation of bone marrow derived mesenchymal stem cells (MSCs) in combination with platelet-rich fibrin glue to full thickness cartilage defects found improvement of symptoms in all patients, with MRI revealing complete defect filling and surface conformity with native cartilage^11^. Davatchi *et al*. reported that patients with moderate to severe OA who were administered autologous MSCs felt a reduction in pain^12^.

Elucidation of the mechanisms governing chondrogenic differentiation of human SSCs offer significant implications for methods to induce novel cartilage formation and potentially aid in the prevention of OA. SSCs have been shown to be regulated by miRNAs including altered chondrogenic differentiation as a consequence of the post-transcriptional regulation of genes involved with the differentiation process^13^,^14^. MiRNAs involved in chondrogenesis could be exploited to induce cartilage regeneration. MiRNAs are single stranded non-coding RNAs which range in length from 20 to 25 nucleotides and regulate gene expression^15^. MiRNAs are processed from longer primary transcripts that undergo processing in the nucleus and cytoplasm to form the small single stranded non-coding RNA^16^. Sequence complementarity between miRNA and its target mRNA determine whether or not the miRNA induces post-transcriptional inhibition or degradation of the mRNA and therefore the prevention of protein translation^17^. This ability of miRNAs to regulate protein translation can allow for the potential exploitation of the function of miRNAs for therapeutic intervention.

Several miRNAs have been shown to modulate chondrogenesis^18^ including cartilage specific miR-140, which is up-regulated during chondrogenic differentiation of human MSCs^19^,^20^. Previously we have examined the expression of miRNAs in regulating human fetal femur-derived SSC differentiation along chondrogenic and osteogenic lineages, identifying miR-146a involvement in regulating TGF-β signalling during chondrocyte development^21^. The first *in vivo* study has shown the combined use of an antisense inhibitor of miR-221 to induce transplanted human MSCs to repair an osteochondral defect^22^. In addition, miRNAs have been found to be aberrantly expressed in OA, suggesting dysregulation in miRNA expression may contribute to or be an indicator of disease pathogenesis^23^. The expression of miR-146a has been shown to be up-regulated in cartilage of patients with low grade OA and postulated to function as an anti-inflammatory mediator by targeting components of intracellular inflammatory signalling including *IRAK1* and *TRAF6* mRNA^24^. Thus miRNAs could be used in combination with SSCs and transplanted to defective articular sites to induce articular cartilage regeneration or directly administered to articular cartilage to modulate resident articular chondrocytes in damaged/diseased cartilage tissue OA.

The current study has examined the role of miR-146b during TGF-β3 induced chondrogenic differentiation of human SSCs. This work demonstrates that miR-146b was down-regulated in human SSCs cultured in the presence of TGF-β3 and that overexpression of miR-146b suppressed SOX5 protein expression. SOX5 is necessary for efficient chondrogenesis and in co-operation with SOX6 enhances the function of the chondrogenic transcription factor SOX9^25^. In addition, we found miR-146b expression was upregulated in chondrocytes isolated from OA articular cartilage, indicating a role for miR-146b in OA pathogenesis. The novel identification of miR-146b down-regulation during chondrogenic differentiation makes miR-146b a favorable target for potential use in future reparative approaches.

## Materials and Methods

### Isolation and culture of human bone marrow derived SSCs

Bone marrow was obtained from patients undergoing total hip replacement surgery at Southampton General Hospital with full ethical consent and approval from the local hospital ethics committee (LREC 194/99/w, 27/10/10) and informed consent was obtained from all subjects. All methods utilising human tissue and cells were performed in accordance within the relevant guidelines and regulations. Bone marrow from 6 individual patients was collected and utilised for the isolation and culture of human bone marrow derived SSCs. Bone marrow was washed and the cell solution passed through a 70 μm cell filter strainer followed by treatment with Lymphoprep™ (Lonza). Isolated mononuclear cells were initialled incubated in blocking buffer (α-MEM, 10% human serum, 5% FCS and 10 mg/ml bovine serum albumin) and then washed with magnetic activated cell sorting (MACS) buffer (BSA and EDTA in PBS). Cells were then incubated in 1 ml of STRO-1 antibody (from hybridoma). Following washing with MACS buffer, cells were re-suspended in 1 ml containing 800 μl MACs buffer and 200 μl rat anti-mouse IgM microbeads (Miltenyi Biotec Ltd). Following washing with MACS buffer target cells were isolated by MACS. Following target cell isolation cells were washed and re-suspended in α-MEM containing 10% FCS and 1% penicillin/streptomycin (P/S) and placed into tissue culture flasks.

### Chondrocyte Isolation

Femoral heads were obtained from patients undergoing total hip replacement surgery at Southampton General Hospital with full ethical consent and approval from the local hospital ethics committee (LREC 194/99/w, 27/10/10) and informed consent was obtained from all subjects. Femoral heads from 22 individual patients; 11 OA femoral heads and 11 femoral heads deemed non-OA were utilised for chondrocyte isolation (See Supplementary Table 1). OA femoral heads were obtained from patients with end stage OA (3–5 OARSI). Femoral heads were not obtained from patients that provided bone marrow samples for the isolation of human bone derived SSCs for use in the isolation of chondrocytes. Articular cartilage was dissected and cut into small pieces within 6 hours of surgery. Cartilage pieces were incubated in 10% trypsin (Sigma Aldrich) for 30 minutes at 37 °C. Following PBS washing of cartilage pieces, cartilage pieces were incubated in 0.1% hyaluronidase (Sigma Aldrich) for 15 minutes, followed by washing and incubation in 1% collagenase B (Roche Diagnostics) in a shaking incubator at 37 °C for 12–15 hours. The digested suspension of articular chondrocytes was filtered through a 70 μm filter. Isolated chondrocytes from 11 NOF (neck of femur breakages) samples (control samples) and 11 OA samples were directly used for extraction of total RNA.

### Chondrogenic micromass differentiation assay

Human bone marrow derived SSCs were seeded at a cell density of 1 × 10^5^ per 10 μl in central spots of individual wells of 24 well plates. 500 μl of α-MEM containing 5% FCS and 1% P/S was carefully added to each well and left overnight. The following day the basal media was removed from the wells and replaced with 500 μl of either chondrogenic media consisting of α-MEM containing 100 μM ascorbate-2-phosphate, 10 nM dexamethasone, 1X ITS liquid media supplement (Sigma) and 10 ng/ml TGF-β3 (Peprotech) or control media consisting of α-MEM and 1X ITS liquid media supplement. Both chondrogenic differentiation media and control media was changed every 48 hours and cells cultured in the micromass system for up to 21 days.

### RNA extraction

Total RNA was extracted from isolated chondrocytes using AllPrep DNA/RNA Mini kit (Qiagen). For all other experiments utilising human bone marrow derived SSCs, total RNA was extracted using mirVana™ RNA Isolation System Kit (Life technologies) in accordance with the manufacturers protocol. In brief samples were washed twice with PBS and 600 μl of lysis buffer was then added to allow for cell membrane lysis and release of RNA. MiRNA homogenizing agent at 10% of the volume of lysis buffer was then added followed by acid phenol-chloroform (Life technologies) to carry out phase separation. The aqueous phase was transferred and added to ethanol followed by spin column based ribonucleic acid purification with use of supplied buffers for washing followed by elution of RNA with RNase free water, followed by RNA quantification with a Thermo Scientific NanoDrop ND-1000 spectrophotometer.

### cDNA synthesis and mRNA expression analysis

cDNA synthesis and qPCR was performed to analyse expression of *SOX9, COL2A1, AGCAN* and *COL9A1* mRNA in human bone marrow derived SSCs following TGF-β3 induced chondrogenesis. cDNA synthesis and qPCR was performed to analyse expression of *MMP13, COL2A1* and *AGCAN* mRNA in human articular chondrocytes. For cDNA synthesis of mRNA in samples, SuperScript^®^ VILO cDNA Synthesis kit was used (Applied Biosystems). In brief, RNA was combined with 2 μl 5X VILO™ reaction mix and 1 μl 10X SuperScript^®^ enzyme and incubated for 10 minutes at 25 °C followed by incubation at 42 °C for 2 hours and 85 °C for a further 5 minutes. qPCR was performed using 10 μl of SYBR-Green master mix, 5 μl of upH_2_O and 2 μl of reverse primer and 2 μl of forward primer for the gene of interest (primers listed in Table 1) and 1 μl of cDNA sample. The final mixture of 20 μl was then added to a 96 well-plate and subsequently analysed with Applied Biosystems, 7500 Real Time PCR system and data produced was analysed with Applied Biosystems 7500 System SDS software, version 2.0.5 program. Standard optimization procedures were carried out to determine the most appropriate housekeeping genes. β-actin, an endogenous housekeeping gene was used to normalise Ct (cross-over threshold) values for SSC experiments and GAPDH was used for experiments which utilised articular chondrocytes. The delta-delta Ct method was used to calculate fold expression levels for each target gene. All reactions were performed in triplicate and included a negative control with no cDNA.

### MiRNA expression analysis

Following RNA extraction samples were analysed for expression of either: miR-146b, miR-140-3p, miR-140-5p or miR-146a using TaqMan^®^ MiRNA Assays (Table 2). Each individual assay contains two primers; one primer for cDNA synthesis and one primer for TaqMan q-PCR. TaqMan^®^ MiRNA Reverse Transcription Kit was used to generate cDNA specific to each assay specific miRNA from total RNA following a modified manufacturer’s protocol. In brief, a reaction mixture was made up of 3.58 μl upH_2_O, 0.75 μl 10X Buffer, 1.88 μl of RNase inhibitor, 1.5 μl of RT primer, μl of dNTPs and 10 ng of total RNA and incubated for 30 minutes at 16 °C followed by 42 °C for 30 minutes and 85 °C for 5 minutes and termination of reaction. qPCR was performed using 5 μl of TaqMan^®^ Universal PCR Master Mix with No AmpErase^®^ UNG (Life technologies) in a reaction mix also containing 3.335 μl of upH_2_O, 0.5 μl of TM primer and 0.8 μl of cDNA. This mix was then transferred to a 96 well-plate and analysed with Applied Biosystems, 7500 Real Time PCR system and data produced was analysed with Applied Biosystems 7500 System SDS software, version 2.0.5. Standard optimization procedures were carried out to determine the most appropriate housekeeping gene for miRNA expression analysis. MammU6, an endogenous RNA housekeeping control for miRNA was used to normalise Ct values for each sample and the delta-delta Ct method was used to calculate fold expression levels for each target gene. All reactions were performed in duplicate and also included a negative control which lacked cDNA.

### Histological analysis

Following 21 days in culture samples were fixed in 4% PFA for 24 h, dehydrated in ethanol washes (50%, 70%, 90% in dH_2_O and 2 × 100%) for 1 hour and incubated in histoclear prior to embedding in paraffin wax. Embedded samples were sectioned at 7 μm thickness. Following slide de-waxing and rehydration, samples were treated with haematoxylin and stained with either Alcian blue or Safranin O or samples were incubated in blocking buffer (1% BSA in PBS) followed by anti-COL2A1 (1:500) (Calbiochem) incubation overnight at 4 °C followed by biotinylated secondary antibody incubation for 1 hour, avidin-conjugated peroxidase treatment and 3-amino-9-ethylcarbazole treatment. Samples were imaged with an Olympus BX-51/22 dotSlide digital virtual microscope using OlyVIA 2.1 software (Olympus Soft Imaging Solutions GmbH).

### Protein extraction and Western Blotting

Following transfection assay human bone marrow derived SSCs were lysed with ~30 μl RIPA buffer (Tris base (Sigma Aldrich), NaCl (Sigma Aldrich) in distilled water adjusted to pH 7.5 with HCl and 10% IGEPAL^®^ CA-630 (Sigma Aldrich), 10% Na-deoxy-cho1ate (Sigma Aldrich), 100 mM EDTA (Fischer Scientific) and 10% SDS (Sigma Aldrich) with added mini protease inhibitor cocktail (Roche)). Cell lysates were then incubated on ice for 20 minutes followed by centrifugation at 13,000 rpm for 20 minutes at 4 °C. The resultant supernatant was collected. The protein concentration of samples was determined using Pierce BCA protein assay kit (Thermo scientific) and 10 μg of each sample combined with DDT and sample loading buffer was analysed by SDS gel electrophoresis and transferred onto polyvinylidene fluoride (PVDF) membrane. Immunoblots were blocked in 1 × PBS, 0.5% tween-20 with 5% non-fat dry milk for one hour at room temperature followed by incubation with rabbit polyclonal anti-SOX5 (1:750) (Abcam) or rabbit polyclonal anti-β-actin (1:500) (Abcam) antibodies overnight at 4 °C. Immunoblots were then washed five times for 5 minutes in 1x PBS, 0.5% tween-20 followed by incubation with Horseradish peroxidase (HRP) conjugated goat anti-rabbit IgG secondary antibody (1:3000) (Abcam) for one hour at room temperature followed by five, 5 minute washes in 1x PBS, 0.5% tween-20. The immunoblot was then incubated in enhanced chemiluminescence (ECL) substrate (Millipore) for 5 minutes followed by chemiluminescent detection with BioRad^®^ Versadoc™ imaging system and densitometry analysis carried out using the BioRad^®^ Quantity One^®^ 4.6.6 software.

### Identifying potential miRNA targets

Target prediction algorithms including TargetScanHuman version 6.0 (http://www.targetscan.org), Diana web server v5.0 interface (http://diana.imis.athena-innovation.gr/DianaTools/index.php?r=microT_CDS/index), PicTar (http://pictar.mdc-berlin.de/) PITA – Segal lab of computational biology (http://genie.weizmann.ac.il/pubs/mir07/mir07_prediction.html) and microRNA.org (Aug 2010 release) (http://www.microrna.org/microrna/home.do) were used to identify potential mRNA targets of miR-146b, which had potential roles in chondrogenesis.

### Transfection Assay

The functional relevance of miR-146b was carried out by transfecting human bone marrow derived SSCs with either miR-146b mimic or a non-targeting negative control miRNA mimic (Thermo Scientific). Human bone marrow derived SSCs were cultured in 6 well plates until confluency followed by transfection with 0.5% DharmaFECT^®^1 in combination with either miR-146b mimic or non-targeting negative control miRNA mimic at final concentrations of 100 nM. After incubation for 48 hours, RNA was extracted for qPCR analysis and protein was obtained for western blotting at 72 hours.

### Statistics

Statistical analysis was performed using GraphPad Prism software version 6.0. Human bone derived SSCs and chondrocytes were obtained from individual subjects. The Wilcoxon signed-rank test was used to compare gene, miRNA and protein expression in all experiments unless otherwise stated. The Friedman test with Dunn’s post-test was used for analysing data from experiments with more than two experimental conditions. The Mann-Witney *U* test was used for comparing gene and miRNA expression between OA chondrocytes and non-OA chondrocytes. *P* values less than 0.05 were considered significant.

## Results

### The expression of miR-146b is down-regulated in TGF-β3 induced chondrogenic differentiation of human bone marrow derived SSCs

**Figure 1:**
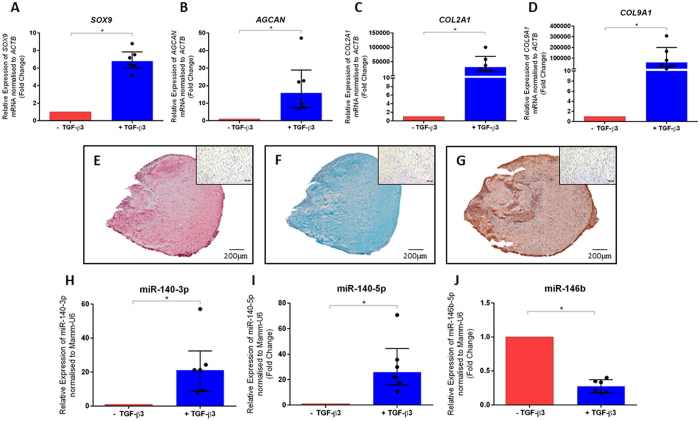
TGF-β3 treatment enhances the expression of chondrogenic associated marker genes, enhances chondrogenic associated histological staining and enhances the expression of chondrogenic associated miR-140-3p and miR-140-5p and decreases the expression of miR-146b in human bone marrow derived SSCs. Data is presented as the median and interquartile quartile range of the fold change in *SOX9* mRNA (**A**), *AGCAN* mRNA (**B**) *COL2A1* mRNA (**C**) *COL9A1* mRNA (**D**), miR-140-3p (**H**), miR-140-5p (**I**) and miR-146b (**J**) expression in human bone marrow derived SSCs cultured in the presence of TGF-β3 for 21 days relative to untreated control bone marrow derived SSCs cultured in the absence of TGF-β3 for 21 days. n = 6, *p < 0.05, Wilcoxon signed rank test. Safranin O stain (**E**), Alcian blue stain (**F**) and type II collagen immuno-staining (**G**) and haematoxylin counterstain in human bone marrow derived SSC micromass cultures after 21 days in the presence and absence of TGF-β3 (inset). Scale bar = 200 μm (**E**–**G**) 100 μm (inset **E**–**G**).

To assess chondrogenic differentiation in SSCs, qPCR was employed to determine the differential expression of chondrogenic marker genes, together with histological staining for chondrogenesis. Human bone marrow derived SSCs seeded at high density as 3D micromass cultures in the presence of TGF-β3 for 21 days exhibited significantly up-regulated expression of chondrogenic associated mRNA; including *SOX9* (5.8-fold change), *AGCAN* (14.9-fold change), *COL2A1* (30,442-fold change) and *COL9A1* (60,827-fold change) expression compared to micromass cultures cultured in the absence of TGF-β3 (Fig. 1A–D).

Following culture for 21 days, micromass cultures were also examined histologically. Human bone marrow derived SSCs cultured in the presence of TGF-β3 formed three-dimensional micromass cultures and exhibited positive safranin O staining (Fig. 1E), alcian blue staining (Fig. 1F) and type II collagen immunostaining (Fig. 1G). Human bone marrow derived SSCs cultured in the absence of TGF-β3 did not form three-dimensional cultures and exhibited negligible staining (Fig. 1E–G insets). The up-regulated expression of chondrogenic marker genes and chondrogenic associated positive histological staining observed in human bone marrow derived SSCs, which had been treated with TGF-β3, indicated differentiation along the chondrogenic lineage.

Following culture of human bone marrow derived SSCs in the presence and absence of TGF-β3 for 21 days, TaqMan qPCR was employed to examine the expression of miRNA. Both miR-140-3p and miR-140-5p served as positive controls for chondrogenic differentiation^20^. Chondrogenic associated miR-140-3p (20.2-fold change) and miR-140-5p (24.8-fold change) expression were both found to be significantly up-regulated in cells treated with TGF-β3 (Fig. 1H–I). Critically, the expression of miR-146b was significantly down-regulated in micromass cultures of human bone marrow derived SSCs cultured in the presence of TGF-β3 compared to cells cultured in the absence of TGF-β3 (0.27-fold change) (Fig. 1J).

**Figure 2:**
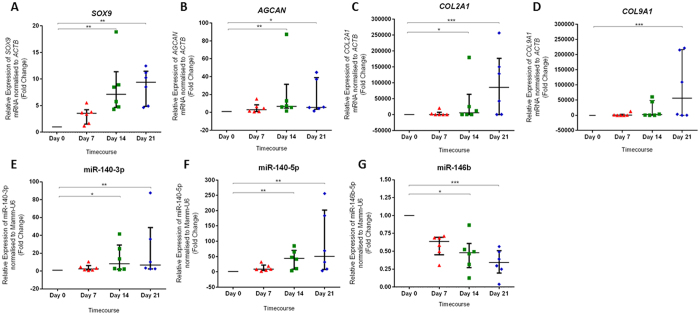
TGF-β3 treatment enhanced chondrogenic marker genes expression and chondrogenic associated miR-140-3p and miR-140-5p expression and decreased miR-146b expression in human SSCs. Data is presented as the median and interquartile quartile range of the fold change in *SOX9* mRNA (**A**), *AGCAN* mRNA (**B**) *COL2A1* mRNA (**C**), *COL9A1* mRNA (**D**), miR-140-3p (**E**), miR-140-5p (**F**) and miR-146b (**G**) expression in human bone marrow derived SSCs cultured in the presence of TGF-β3 for up to 21 days relative to untreated control human bone marrow derived SSCs at day 0. mRNA and miRNA expression was analysed at even time points across 21 days at days 0, 7, 14 and 21. n = 6, *p < 0.05, **p < 0.01, ***p < 0.001, Friedman test with Dunn’s post-test.

To observe the temporal effect of TGF-β3 on human bone marrow derived SSCs across a period of 21 days, qPCR and TaqMan qPCR was employed to examine chondrogenic marker gene expression and miRNA expression at even time points at days 0, 7, 14 and 21. Human bone marrow derived SSCs cultured in the presence of TGF-β3 displayed up-regulated expression of *SOX9, AGCAN, COL2A1* and *COL9A1* mRNA (Fig. 2A–D). *SOX9* (6.10, 8.39-fold change), *AGCAN* (5.68, 4.70-fold change) and *COL2A1* (5,903, 85,456-fold change) mRNA exhibited significant upregulation in expression at day 14 and day 21 compared to expression at day 0 (Fig. 2A–C). *COL9A1* mRNA expression was significantly up-regulated at day 21 compared to expression at day 0 (56,159-fold change) (Fig. 2D). Human bone marrow derived SSCs cultured in the presence of TGF-β3 exhibited significant upregulation in expression of miR-140-3p (7.36, 5.80-fold change) and miR-140-5p (43.11, 49.36-fold change) at day 14 and day 21 compared to the expression at day 0 (Fig. 2E,F). Cells exhibited significant down-regulation in miR-146b expression at days 14 and 21 compared to the expression at day 0 (0.48, 0.31-fold change) (Fig. 2G).

### The expression of SOX5 is down-regulated in response to treatment of human bone marrow derived SSCs with miR-146b mimic

The expression of miR-146b was significantly down-regulated following TGF-β3 induced chondrogenic differentiation of human bone marrow SSCs. To further examine miR-146b down-regulation in these cells, mRNA target prediction programmes (TargetScanHuman, Diana, PicTar, microRNA.org) were employed to identify potential chondrogenic associated mRNA targets of miR-146b. *SOX5* mRNA was listed as a potential miR-146b target by all of the target prediction algorithms. Identification of a potential miR-146b target enabled the use of miR-146b mimic to increase the level of miR-146b within human bone marrow derived SSCs to determine the effect upon *SOX5* mRNA and SOX5 protein expression.

**Figure 3:**
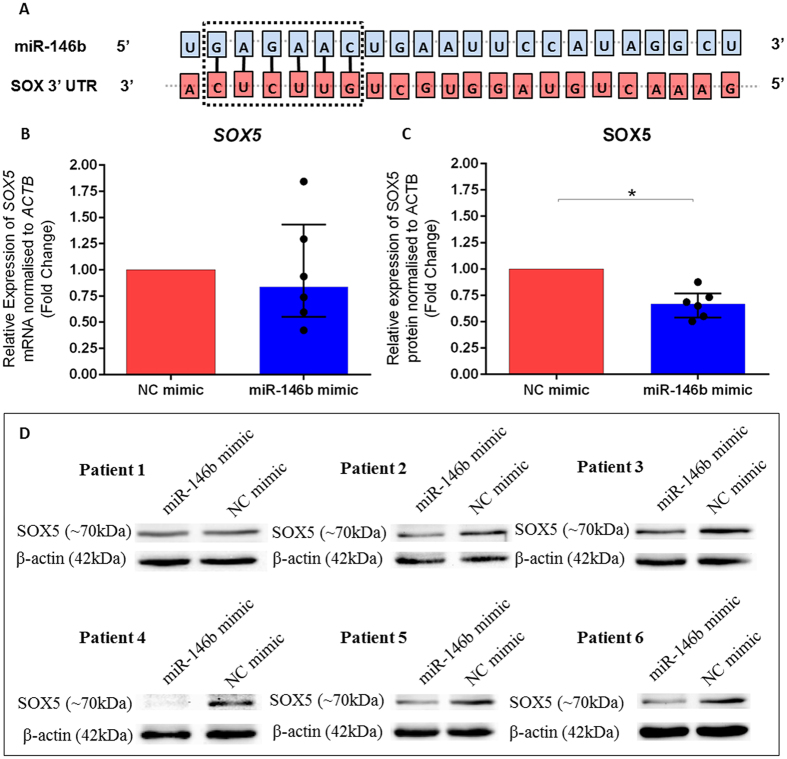
miR-146b mimic reduces SOX5 protein expression in human bone marrow derived SSCs. Seed match base pairing of mature miR-146b seed region to the designated sequence within *SOX5* mRNA. (Information regarding miRNA sequences and targets were interpreted from TargetScanHuman, version 6.0) (**A**). Data is presented as the median and interquartile quartile range of the fold change in *SOX5* mRNA (**B**) and SOX5 protein (**C**) expression in human bone marrow derived SSCs cultured in the presence miR-146b mimic relative to control cells treated with non-targeting miRNA mimic. n = 6, *p < 0.05, Wilcoxon signed rank test. Immunoblots from 6 individual patient samples (**D**) were used for determination of SOX5 protein expression using densitometry analysis and β-actin was used as the normalisation control. Cropped blots have been presented. Full length blots are presented in Supplementary Figure S1.

Human bone marrow derived SSCs were transfected with miR-146b mimic or a miRNA mimic non-targeting control for both mRNA and protein expression analysis. Following transfection, *SOX5* mRNA expression was analysed using qPCR and SOX5 protein expression determined using western blotting and densitometry analysis. Transfection of miR-146b mimic did not affect the expression of *SOX5* mRNA significantly, when compared to cells transfected with miRNA mimic non-targeting control (Fig. 3B). However, when protein levels were evaluated a significant down-regulation of SOX5 protein was observed (0.67-fold change) (Fig. 3C). Immunoblots confirmed the down-regulation of SOX5 expression from 6 individual human bone marrow derived SSCs patient samples following transfection with miR-146b mimic or miRNA mimic non-targeting control (Fig. 3D).

### The expression of miR-146b is up-regulated in chondrocytes isolated from human OA articular cartilage

**Figure 4:**
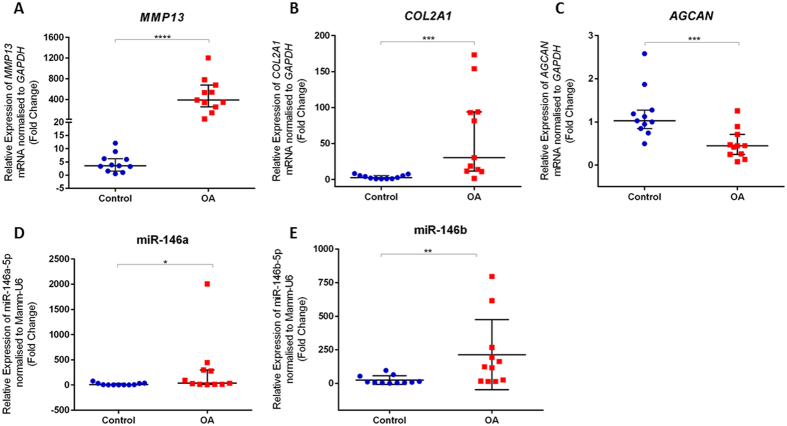
Dysregulated mRNA, miR-146a and miR-146b expression is observed in OA articular chondrocytes compared to control chondrocytes. Data presented as the median and interquartile range of the fold change in *MMP13* mRNA (**A**), *COL2A1* mRNA (**B**) and *AGCAN* mRNA (**C**), miR-146a (**D**) and miR-146b (**E**) expression in OA articular chondrocytes relative to control chondrocytes. Values are presented as individual biological replicates n = 11, *P < 0.05, **P < 0.01, ***P < 0.001, ****P < 0.0001, Mann-Whitney U test. Supplementary Figure S1. Full length images of the cropped blots presented in main Fig. 3D. Full length images in Figure S1 demonstrate that increased levels of miR-146b in human bone marrow derived SSCs reduces SOX5 expression. Human bone marrow derived SSCs were cultured in the presence of miR-146b mimic and non-targeting miRNA mimic. Human bone marrow derived SSCs were isolated from 6 individual patient samples. β-actin was used as the internal control.

To determine whether the expression of miR-146b is dysregulated in OA, the relative expression of miR-146b between OA chondrocytes isolated from OA articular cartilage and chondrocytes from non-OA cartilage was examined. Chondrocytes isolated from OA articular cartilage exhibited significant up-regulated expression of *MMP13* mRNA (386.8-fold change) and *COL2A1* mRNA (27.7-fold change) (Fig. 4A,B) and significant down-regulated expression of *AGCAN* mRNA (0.58-fold change) (Fig. 4C). TaqMan qPCR identified significant up-regulated expression of OA associated miR-146a (28.14-fold change) (Fig. 4D). Critically, the expression of miR-146b was found to be significantly up-regulated in chondrocytes isolated from OA articular cartilage, these levels were 115.4-fold higher than those observed in non-OA chondrocytes (Fig. 4E).

## Discussion

In this study we show that the expression of miR-146b is progressively decreased during chondrogenic differentiation of human bone marrow derived SSCs. The seed region of miR-146b has been identified through bioinformatic approaches to base pair with nucleotides within the 3′UTR of *SOX5* mRNA. Overexpression of miR-146b following transfection of human bone marrow derived SSCs resulted in the down-regulation of SOX5. In this study, the down-regulated expression of miR-146b observed during TGF-β3 induced chondrogenic differentiation is likely to enable SOX5 expression de-repression. The progressive downregulation of miR-146b expression during chondrogenesis, overrides the inhibitory effect of miR-146b upon SOX5 expression, which facilitates chondrogenic differentiation of human bone marrow derived SSCs.

Previous studies have demonstrated that miR-146b has a critical role in differentiation. The expression of miR-146b has been shown to be up-regulated during myoblast differentiation and muscle regeneration *in vivo* acting as a positive regulator of myogenesis in mice^26^. The expression of miR-146b has also been shown to be up-regulated in hematopoietic stem/progenitor cells that underwent erythroid or megakaryocytic differentiation, miR-146b was found to directly target a negative regulator of erythroid and megakaryocyte differentiation^27^. For the first time, miR-146b has also been identified to play a role in the chondrogenic differentiation of human bone marrow derived SSCs, as a negative regulator of chondrogenesis, through modulation of SOX5.

*SOX5* encodes the transcription factor SOX5. In chondrogenesis SOX5 is co-expressed with SOX9 and SOX6. It is thought that SOX5 and SOX6 form homo and heterodimers which co-operate with SOX9 to enhance chondrogenic associated gene up-regulation^28^. SOX5 along with SOX6 and SOX9 has been shown to bind to the enhancer region of *COL2A1* and co-expression of all SOX proteins was shown to induce higher expression of *COL2A1*^29^. Han *et al*. have shown that both SOX5 and SOX6 are required for the binding of SOX9 to the *AGC1, COL2A1* and *COL11A1* enhancers^30^. In a murine study pre-chondroblasts from *Sox5*^−/−^; *Sox6*^−/−^ double null embryos failed to differentiate into chondroblasts and expressed low levels of cartilage matrix components^31^. Sox5 has also been shown to co-operate with Sox6 and Sox9 to induce chondrogenic associated miR-140 expression, with identification of a Sox5/Sox6/Sox9 response element in the upstream region of miR-140^32^. Given the positive role that *SOX5* has during chondrogenesis, *SOX5* levels must be regulated to ensure optimal functioning, miR-146b acts as a negative regulator of *SOX5*. This current study has shown that transfection of miR-146b mimic increased levels of miR-146b and down-regulated SOX5 expression, delineating miR-146b as anti-chondrogenic. Similar to the effect of increased levels of miR-146b on SOX5 expression, down-regulation of SOX5 has previously been demonstrated in human adipose-derived stem cells as a result of miR-194 overexpression. Like miR-146b, miR-194 was also found to be down-regulated during chondrogenesis^33^. Furthermore, the role of a network of microRNAs enhancing the effect of miR-146b by affecting interconnected pathways cannot be ruled out. For instance, miR140 and miR-193b, both associated with chondrogenic development can also target Wnt and TGF-β signalling^34^,^35^, pathways that interact with the SOX transcription factor family in chondrogenesis.

Repairing initial articular cartilage defects may be a propitious option for halting further cartilage degradation and potentially preventing the onset of OA. OA joints lack healthy functional chondrocytes and exhibit deterioration of the cartilage ECM^36^ and absence of endogenous articular cartilage regeneration. Human bone marrow derived SSCs, with cartilage differentiation capacity, in combination with select miRNAs known to regulate chondrogenic differentiation therefore could aid chondrogenesis. MiRNAs have functions in maintaining the homeostatic balance in articular chondrocytes and cartilage and aberrant signaling may impact upon miRNA expression which may induce changes in expression of genes involved with maintaining articular cartilage integrity^23^,^37^. Indeed, miRNA expression signatures have been associated with distinct pathological features in OA disease progression^23^, with miRNAs observed to be differentially expressed in OA cartilage tissue compared to normal cartilage tissue^38^ and in chondrocytes isolated from OA cartilage compared to chondrocytes isolated from normal cartilage^39^.

The expression of *MMP13, COL2A1* and *AGCAN* mRNA in chondrocytes isolated from the articular cartilage of OA patients reaffirmed that the cartilage samples were of OA origin. The expression of OA associated *MMP13* mRNA, which encodes MMP13 and degrades type II collagen, was found to be significantly up-regulated in chondrocytes isolated from the articular cartilage of OA patients, consistent with previous findings^40^,^41^. The expression of *COL2A1* mRNA was significantly elevated in chondrocytes isolated from the articular cartilage of OA patients. The expression of *COL2A1* has been previously shown to be significantly up-regulated in chondrocytes isolated from high grade OA articular cartilage and proposed to be as a result of potential anabolic response by chondrocytes to restore the ECM^42^ and microarray analyses have also identified increased *COL2A1* expression in OA^43^,^44^. The expression of miR-146b was significantly elevated in chondrocytes isolated from the articular cartilage of OA patients, indicating miR-146b expression is dysregulated in OA and therefore a potential contributor or indicator of the underlying pathophysiology of OA. MiR-146b may therefore serve as a potential therapeutic target in the treatment of OA. However, the function of miR-146b in human articular chondrocytes remains to be elucidated.

The expression of miR-146a has previously been shown to be up-regulated in OA and has been suggested to target pro-inflammatory mediators^24^. Yamasaki *et al*. identified that miR-146a expression increased in chondrocytes beginning to undergo degenerative changes and that the prominent OA cytokine IL-1β induced the expression of miR-146a in normal chondrocytes^24^. MiR-146a has also been found to up-regulated in an OA rat model^45^ and miR-146a has also been shown to be up-regulated in mechanically injured human chondrocytes^46^. However, in addition to studies which have observed the up-regulation of miR-146a in OA, important to mention, in contrast, a study which identifies down-regulation of miR-146a in OA chondrocytes^47^. In several human pathological disorders involving inflammatory response activation, miR-146a and miR-146b have been found to be up-regulated^48^,^49^,^50^,^51^,^52^,^53^,^54^,^55^,^56^,^57^,^58^,^59^. MiR-146b shares close sequence homology to miR-146a and therefore miR-146b could also be up-regulated in OA and target pro-inflammatory mediators acting as an anti-inflammatory mediator, similar to the suggested function of the closely related miR-146a.

In conclusion, the current studies demonstrated that miR-146b is down-regulated during the chondrogenic differentiation of human bone marrow derived SSCs. Increased miR-146b levels was accompanied by SOX5 down-regulation in human bone marrow derived SSCs. SOX5 is critical for efficient early chondrogenic differentiation^31^,^60^. The expression of miR-146b was also found to be up-regulated in OA, suggesting a role in the disease pathogenesis and may serve as a potential direct therapeutic target. To our knowledge, this is the first study that identifies the expression and functional relevance of miR-146b in the chondrogenic differentiation of human stem cells. Modulation of miR-146b expression in isolated human bone marrow derived SSCs may provide a novel technique for enhancing chondrogenic differentiation and cartilage regeneration at sites of articular cartilage injury and thus potentially prevent the onset of OA. These findings indicate the importance of delineating the role of miR-146b within an *in vivo* study on human bone marrow SSCs differentiation capacity in a chondral defect to confirm the therapeutic potential of this miRNA.

## Additional Information

**How to cite this article**: Budd, E. *et al*. MiR-146b is down-regulated during the chondrogenic differentiation of human bone marrow derived skeletal stem cells and up-regulated in osteoarthritis. *Sci. Rep.* **7**, 46704; doi: 10.1038/srep46704 (2017).

**Publisher's note:** Springer Nature remains neutral with regard to jurisdictional claims in published maps and institutional affiliations.

## Supplementary Material

Supplementary Information

## Figures and Tables

**Table 1:** Primer sequences for genes examined in and corresponding amplicon size.

Gene	Primer Sequences	Amplicon size
Human *ACTB*	F: 5′ggcatcctcaccctgaagta 3′ R: 5′aggtgtggtgccagattttc 3′	82 bp
Human *SOX9*	F: 5′cccttcaacctcccacacta 3′ R: 5′ tggtggtcggtgtagtcgta 3′	74 bp
Human *COL2A1*	F: 5′cctggtccccctggtcttgg 3′ R: 5′ catcaaatcctccagccatc 3′	58 bp
Human *ACGAN*	F: 5′gacggcttccaccagtgt 3′ R: 5′gtctccatagcagccttcc 3′	90 bp
Human *COL9A1*	F: 5′cctggtgctcttggtttga 3′ R: 5′ cacgctcccccttttctc 3′	58 bp
Human *MMP13*	F: 5′ttaaggagcatggcgacttct 3′ R: 5′ cccaggaggaaaagcatgag 3′	71bp
Human *SOX5*	F: 5′tagctagtccttcagccagagtt 3′ R: 5′ccttcatttgccgagcttctt 3′	93 bp

**Table 2:** TaqMan^®^ MiRNA Assays used for miRNA expression analysis from Applied Biosystems, Life Technologies.

MiRNA	TaqMan MiRNA Assay Name	Assay ID number
Mamm U6 – U6 spliceosome RNA	U6 snRNA	001973
miR-140-5p	mmu-miR-140-5p	001187
miR-140-3p	hsa-miR-140-3p	002234
miR-146a-5p	hsa-miR-146a	00468
miR-146b-5p	hsa-miR-146b	001097
