# Supplementary material for: MiR-146b is down-regulated during the chondrogenic differentiation of human bone marrow derived skeletal stem cells and up-regulated in osteoarthritis
Source: Sci Rep. 2017 Apr 24;7:46704. doi: 10.1038/srep46704 (PMC5402270; doi:10.1038/srep46704)
Supplement: Supplementary Information [file srep46704-s1.pdf]

Supplementary Information

MiR-146b is down-regulated during the chondrogenic differentiation of human bone marrow derived skeletal stem cells and up-regulated in osteoarthritis

Emma Budd[1], María C. de Andrés[1], Tilman Sanchez-Elsner[2], Richard OC Oreffo[1]

[1]Bone and Joint Research Group, Centre for Human Developmental, Stem Cells and Regeneration, Faculty of Medicine, University of Southampton.

[2] Junk RNA group, Clinical and Experimental Sciences, Faculty of Medicine, University of Southampton.

Sample	Gender	Age		Sample	Gender	Age	
1OA	M	69		1NOF	F	95	
2OA	M	72		2NOF	F	78	
3OA	M	46		3NOF	F	86	
4OA	F	52		4NOF	F	81	
5OA	F	61		5NOF	F	85	
6OA	M	70		6NOF	M	85	
7OA	M	66		7NOF	M	89	
8OA	F	88		8NOF	F	83	
9OA	F	75		9NOF	F	67	
10OA	M	77		10NOF	F	89	
11OA	M	73		11NOF	M	73	
n= 11	36.4% F	68.1	Mean	n= 11	72.7% F	82.8	Mean
	63.6% M	11.7	SD		27.3% M	7.9	SD

Supplementary Table 1. Femoral heads from 22 individual patients; 11 OA femoral heads and 11 femoral heads deemed non-OA were utilised for chondrocyte isolation. The articular cartilage samples utilised in the study were selected at random. OA femoral heads were obtained from patients with end stage OA (3-5 OARSI).

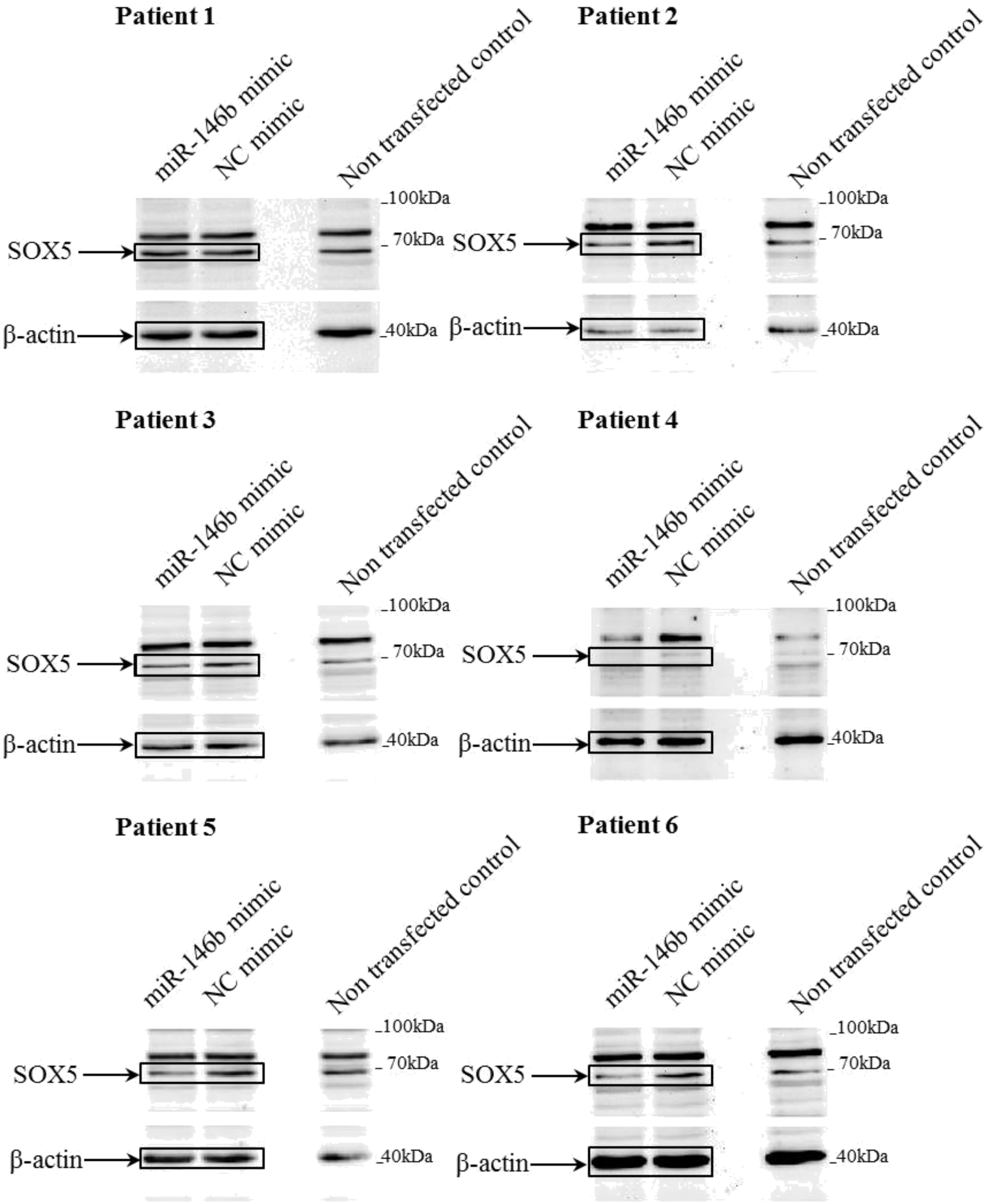

Supplementary Figure S1. Full length images of the cropped blots presented in main Figure 3D. Full length images in Figure S1 demonstrate that increased levels of miR-146b in human bone marrow derived skeletal stem cells reduces SOX5 expression. Human bone marrow derived SSCs were cultured in the presence of miR-146b mimic and non-targeting miRNA mimic. Human bone marrow derived SSCs were isolated from 6 individual patient samples.

β-actin was used as the internal control.
